# Perturbation of Human Coronary Artery Endothelial Cell Redox State and NADPH Generation by Methylglyoxal

**DOI:** 10.1371/journal.pone.0086564

**Published:** 2014-01-21

**Authors:** Philip E. Morgan, Pamela J. Sheahan, Michael J. Davies

**Affiliations:** 1 Free Radical Group, The Heart Research Institute, Sydney, New South Wales, Australia; 2 Faculty of Medicine, University of Sydney, Sydney, New South Wales, Australia; Case Western Reserve University, United States of America

## Abstract

Diabetes is associated with elevated plasma glucose, increased reactive aldehyde formation, oxidative damage, and glycation/glycoxidation of biomolecules. Cellular detoxification of, or protection against, such modifications commonly requires NADPH-dependent reducing equivalents (*e.g.* GSH). We hypothesised that reactive aldehydes may modulate cellular redox status *via* the inhibition of NADPH-generating enzymes, resulting in decreased thiol and NADPH levels. Primary human coronary artery endothelial cells (HCAEC) were incubated with high glucose (25 mM, 24 h, 37°C), or methylglyoxal (MGO), glyoxal, or glycolaldehyde (100–500 µM, 1 h, 37°C), before quantification of intracellular thiols and NADPH-generating enzyme activities. Exposure to MGO, but not the other species examined, significantly (*P*<0.05) decreased total thiols (∼35%), further experiments with MGO showed significant losses of GSH (∼40%) and NADPH (∼10%); these changes did not result in an immediate loss of cell viability. Significantly decreased (∼10%) NADPH-producing enzyme activity was observed for HCAEC when glucose-6-phosphate or 2-deoxyglucose-6-phosphate were used as substrates. Cell lysate experiments showed significant MGO-dose dependent inhibition of glucose-6-phosphate-dependent enzymes and isocitrate dehydrogenase, but not malic enzyme. Analysis of intact cell or lysate proteins showed that arginine-derived hydroimidazolones were the predominant advanced glycation end-product (AGE) formed; lower levels of *N* ^ε^-(carboxyethyl)lysine (CEL) and *N* ^ε^-(carboxymethyl)lysine (CML) were also detected. These data support a novel mechanism by which MGO exposure results in changes in redox status in human coronary artery endothelial cells, *via* inhibition of NADPH-generating enzymes, with resultant changes in reduced protein thiol and GSH levels. These changes may contribute to the endothelial cell dysfunction observed in diabetes-associated atherosclerosis.

## Introduction

Diabetes is a complex disease defined by elevated blood glucose and decreased insulin production or sensitivity. People with diabetes have a >2-fold increased risk of developing atherosclerosis and dying from cardiovascular disease compared to those without diabetes [1]. The increased levels of blood glucose and ketones in people with diabetes result in elevated levels of reactive aldehydes, including methylglyoxal (MGO), glyoxal and glycolaldehyde (reviewed in [2], [3]). These arise from multiple non-enzymatic (autoxidation/glycation/glycoxidation) and metabolic pathways, including triose phosphate metabolism [3]. Normal steady-state plasma levels of MGO (*i.e.* the unreacted concentration at a specific time point) are generally accepted to be nanomolar in healthy controls (though considerably higher concentrations have also been reported [4]), with significant elevations observed in both Type 1 and Type 2 diabetes patients, and particularly in ketosis, sometimes to micromolar levels [5] (reviewed in [4]). Similarly, unreacted plasma levels of glyoxal are typically nanomolar, but can reach micromolar levels in people with diabetes [6], [7]. Glycolaldehyde has been implicated as an important reactive intermediate in *in vitro* studies (reviewed in [8]), however its high reactivity with biological targets has precluded measurement of plasma levels.

**Figure 1:**
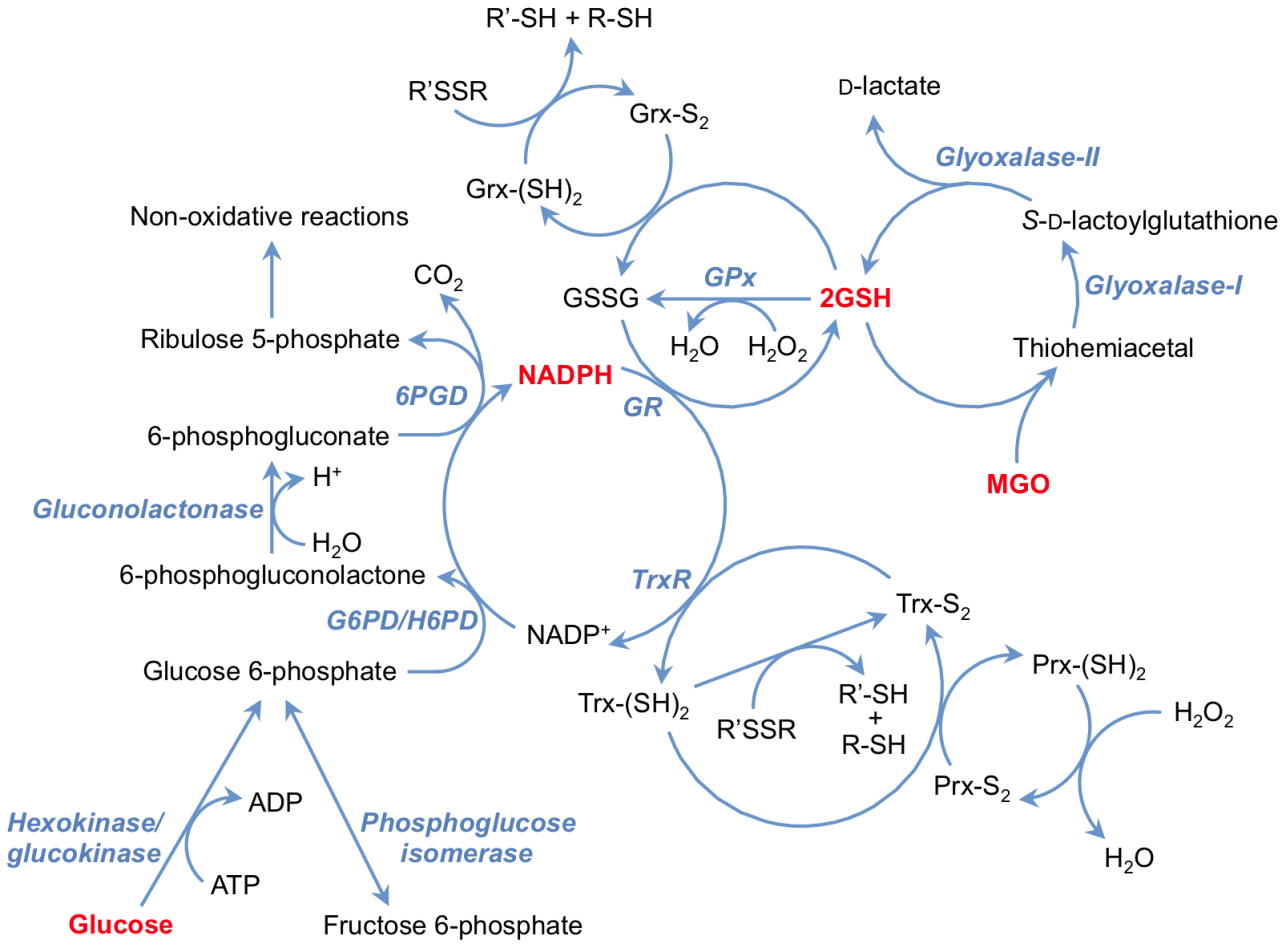
Interconnection of the oxidative pentose phosphate pathway with antioxidant enzymes and the glyoxalase system. Abbreviations: glutathione, GSH; glutathione peroxidase, GPx; thioredoxin, Trx; thioredoxin reductase, TrxR; peroxiredoxin, Prx; glutaredoxin, Grx. Modified from [45].

Reactive aldehydes are detoxified *via* their rapid, spontaneous reaction with glutathione (GSH) to yield thiohemiacetals. In the case of MGO (Figure 1; top right), its thiohemiacetal is converted to *S*-d-lactoyl-glutathione by cytosolic glyoxalase I. The *S*-d-lactoyl-glutathione is then converted to d-lactate and GSH by glyoxalase II. The glyoxalase system can also metabolise other reactive aldehydes, including glyoxal, hydroxypyruvaldehyde and 4,5-dioxovalerate (reviewed in [9]). The glyoxalase system requires GSH, and its rate of reactive aldehyde detoxification can be limited by GSH depletion. Recycling of oxidised GSH (GSSG) occurs *via* the glutathione reductase/NADPH system. Many other protective enzymes are also dependent on GSH and NADPH, including glutathione peroxidases (GPx, which remove H_2_O_2_, and in some cases lipid hydroperoxides), glutaredoxins and thioredoxins (Grx and Trx, which reduce protein disulphides), and peroxiredoxins (Prx, which remove peroxides) (Figure 1).

NADPH is produced predominantly in the cytosol by the pentose phosphate pathway, *via* glucose-6-phosphate dehydrogenase (G6PD) and 6-phosphogluconate dehydrogenase (6PGD; Figure 1). NADPH is also produced to a lesser extent by isocitrate dehydrogenase (IDH) and malic enzyme (ME) in both the cytosol and mitochondria, whilst hexose-6-phosphate dehydrogenase (H6PD) supplies NADPH for cortisol regeneration within the endoplasmic reticulum [10]. Since NADPH is required for these protective systems, perturbations in NADPH generation would be expected to modulate the cellular redox environment, and result in increased oxidation and glycation and modified cell function and survival.

The glyoxalase and other aldehyde removal systems (*e.g.* aldose reductase and 2-oxoaldehyde dehydrogenase [11]) are not completely effective, with elevated levels of advanced glycation end-products (AGEs - a heterogeneous group of compounds formed on reaction of glucose and aldehydes with nucleophiles present on DNA and proteins [3]) detected in organs and tissues that are affected by diabetes (reviewed [12]). Although the consequences of AGE formation are not fully elucidated, they can accumulate in cells and on matrix proteins [13], modulate enzyme activity [14], modify protein turnover [15], [16] and affect cell signalling (particularly *via* RAGE and other receptors [17]).

Endothelial cells are particularly susceptible to hyperglycaemia-induced damage due to their ready exposure to elevated glucose and aldehyde levels, and endothelial dysfunction is an early and defining feature of cardiovascular disease. We therefore hypothesised that elevated glucose and reactive aldehydes can modulate the redox balance of endothelial cells *via* inhibition of NADPH-generating enzymes, resulting in decreased reduced thiol and NADPH levels. This has been investigated in primary human coronary artery endothelial cells (HCAEC) from multiple donors, and we show here that incubation of HCAEC with MGO concentrations relevant to poorly controlled diabetes or ketosis results in decreased total reduced thiols, GSH and NADPH, with an accompanying decrease in the activities of several NADPH-producing enzymes.

## Materials and Methods

### Ethics Statement

The cells used in this study were obtained by the supplier with documented informed donor consent, and analysed anonymously.

### Materials

Chemicals were from Sigma-Aldrich (Castle Hill, NSW, Australia) unless noted. Water was from a Millipore (North Ryde, NSW, Australia) Milli Q Advantage A10 system. HPLC/UHPLC solvents were from EMD (Kilsyth, Vic, Australia). Trace metal ions were removed by treatment with washed Chelex-100 resin (Bio-Rad, Gladesville, NSW, Australia). The concentration of the stock MGO solution (∼40% aqueous solution) was checked using the *N*-acetyl-l-cysteine assay [18] as reported previously [19]. Levels of formaldehyde in the stock MGO solution were also checked in the light of concerns in some publications (reviewed in [20]); UHPLC analysis of its 2,4-dinitrophenylhydrazine (DNPH) derivative [21] revealed that formaldehyde comprised ∼1.4% of the solution.

### Cell Culture

Primary human coronary artery endothelial cells (HCAEC; purchased from Cell Applications, San Diego, CA, USA) were cultured (37°C, 5% CO_2_) in MesoEndo medium (Cell Applications). Cell viability was determined by lactate dehydrogenase (LDH) release [16]. Cells were passaged at ∼80% confluence, and used from passages 4–8. Cells were detached using trypsin-EDTA (Thermo Fisher Scientific, Scoresby, Victoria, Australia). For intact cell experiments, cells were diluted with 10 mL medium to inactivate trypsin, pelleted (5 min, 930×*g*, 21°C) and resuspended at 1–1.5×10^5^ cells/mL. Aliquots (2 mL) were transferred to 6-well plates and incubated overnight before use. For lysate studies, trypsinised cells were washed twice in PBS (Astral Scientific, Caringbah, NSW, Australia), and then lysed in water (2×10^7^ cells/mL) prior to freezing at −80°C.

### Exposure of HCAEC to Reactive Aldehydes and High Glucose

Intact HCAEC (2–3×10^5^ cells/well) were washed with HBSS, followed by incubation (1 h, 37°C, 5% CO_2_) with MGO, glyoxal or glycolaldehyde (0–500 µM) in HBSS without Mg^2+^ or Ca^2+^, to avoid reaction with medium proteins. Incubations using 25 mM glucose were carried out for 24 h (37°C, 5% CO_2_), by addition of d-glucose (Merck, Kilsyth, Victoria, Australia) to the medium. Cells were subsequently washed (HBSS) and lysed using phosphate buffer (50 mM, pH 7, 4°C) containing 10 mM EDTA. Lysates (1–2 mg protein/mL) were incubated with MGO (0–5 mM, 3 h, 37°C) in PBS.

### Bicinchoninic Acid (BCA) Protein Assay

Where no residual MGO was present (*i.e.* intact HCAEC incubated pre-lysis, or washed lysates), protein concentrations of the lysed cells were determined by BCA assay (Pierce, Rockford, IL, USA). Lysates (100 µL) were diluted to 250 µL with 50 mM phosphate buffer (pH 7, containing 10 mM EDTA), then mixed with 2 mL of BCA working reagent. Following incubation (1 h, 60°C), the absorbance of 200 µL of room temperature sample was determined at 562 nm. BSA standards (0–0.25 mg/mL) were run in parallel.

### Bradford Protein Assay

Where HCAEC lysates were incubated with MGO post-lysis and unreacted MGO may still have been present, protein concentrations were determined using the Bradford assay, using 10 µL of diluted samples and 200 µL of Bradford reagent (Bio-Rad). Following incubation (5 min, 21°C), absorbance at 595 nm was measured. BSA standards (0–1 mg/mL) were run in parallel.

### Quantification of Cellular Thiols

Thiol concentrations were determined by incubation (1.5 h, 37°C, in darkness) of 980 µL of lysates with 20 µL of 10 mM 5,5′-dithio-bis(2-nitrobenzoic acid) (DTNB), with subsequent measurement of absorbance at 412 nm [22]. Data were standardised to protein levels as determined by BCA protein assay.

### Quantification of GSH and GSSG

GSH and GSSG were quantified using a GSH-Glo™ Glutathione Assay Kit (Promega, Alexandria, NSW, Australia) according to the manufacturers’ instructions, following incubation of 1×10^4^ cells/well in 96-well plates (1 h, 37°C, 5% CO_2_) with 0–500 µM MGO in serum-free HBSS with added Ca^2+^ and Mg^2+^. For total glutathione determinations, GSSG and protein-SSG were reduced to GSH using 500 µM Tris(2-carboxyethyl)phosphine hydrochloride before analysis.

### Quantification of NADP^+^/NADPH

HCAEC (3×10^5^ per well) were incubated (1 h, 37°C, 5% CO_2_) in HBSS with 0 or 500 µM MGO. After washing twice with HBSS, and freeze-thawing in extraction buffer, total (NADP^+^+NADPH), and NADPH alone, were quantified using a NADP^+^/NADPH kit (BioVision, Mountainview, CA, USA), according to the manufacturers’ instructions.

### Enzyme Assays for NADPH-producing Enzymes

Enzyme activities (1–2 mg protein/mL, 240 µL final volume, normalised for protein content by Bradford assay) were assessed at 37°C over 20 min. Residual aldehyde was not removed prior to assay as pilot studies showed no interference (data not shown). For G6P utilising enzymes (G6PD +6PGD+H6PD) reaction mixtures contained 25 mM Tris-HCl (pH 7.4), 5 mM MgCl_2_, 5 mM G6P (Merck) and 0.5 mM NADP^+^ (Roche). H6PD activity was determined using 2-deoxyglucose-6-phosphate (dG6P). The combined contribution of G6PD +6PGD+H6PD was assessed using fructose-6-phosphate (F6P), whereby phosphoglucose isomerase initially converts F6P to G6P. IDH activity was measured with 25 mM Tris-HCl (pH 7.4), 5 mM MgCl_2_, 5 mM isocitrate and 0.5 mM NADP^+^. ME activity was measured with 25 mM Tris-HCl (pH 7.4), 5 mM MgCl_2_, 5 mM l-malic acid disodium salt (Research Organics, Cleveland, OH, USA) and 0.5 mM NADP^+^.

### AGE Analysis by ELISA

Intact HCAEC (3×10^5^ per well; from 4 donors) and HCAEC lysates (1 mg/mL; from 4 donors) were incubated with MGO as above. Intact cells were washed twice with HBSS following incubation, then lysed with 100 µL of water, removed from the wells by scraping, and frozen at −80°C until used. For lysate incubations, any unreacted MGO was removed immediately following incubation by 3 water washes/concentration cycles using 10 kDa MWCO centrifugal concentrators (Pall Nanosep 10 K Omega; 14000×*g*), followed by freezing at −80°C until needed. AGEs were quantified using the following kits (all from Cell Biolabs, Inc., San Diego, CA, USA) as per the manufacturers’ instructions: OxiSelect™ Methylglyoxal Competitive ELISA kit, to detect hydroimidazolone formation; OxiSelect™ *N* ^ε^-(carboxyethyl)lysine (CEL) Competitive ELISA Kit, to detect CEL; and OxiSelect™ *N* ^ε^-(carboxymethyl)lysine (CML) Competitive ELISA kit, to detect CML. In all cases 50 µL of 0.3 mg/mL protein (adjusted with water where necessary) was loaded per well. Cells incubated with MGO whilst intact were assayed separately for each of the 4 donors. Cells incubated with MGO post-lysis were pooled to give equal amounts of protein from each donor, then assayed in triplicate. Quantification of the products formed was by comparison to standards supplied with each kit (hydroimidazolone, CEL or CML, respectively); cross-reactivity of the anti BSA-hydroimidazolone antibody with BSA-CEL or BSA-CML is reported to be <0.001%.

### Acid Hydrolysis and HPLC Analysis of HCAEC Lysates

Lysates (1 mg protein/mL; each replicate from a different donor) were incubated with MGO (0 or 5 mM, 3 h, 37°C, in 10 mM phosphate buffer, pH 7.0, containing 2 mM EDTA). Proteins (0.25 mg protein/vial) were then precipitated, washed, and hydrolysed with methanesulfonic acid (MSA) [22]. HPLC analysis of *o*-phthaldialdehyde (OPA) derivatised AGEs was performed using a Shimadzu LC-10 HPLC system (Shimadzu Scientific Instruments, Rydalmere, NSW, Australia) as previously [23], with all samples filtered through 0.2 µm centrifugal filters (Pall Nanosep MF; 2 min, 9300×*g*) prior to analysis. Samples used 40 µL injections; AGE standards used 30 µL injections. MGO-derived AGEs were quantified using the following standards (all from Polypeptide Laboratories, Strasbourg, France): *N* ^ε^-(carboxymethyl)lysine (CML); *N* ^ε^-(carboxyethyl)lysine (CEL), (2*S*)-2-amino-5-(5-methyl-4-oxo-4,5-dihydro-1*H*-imidazol-2-ylamino)-pentanoic acid (MG-H1), and (2*S*)-2-amino-5-(2-amino-5-methyl-4-oxo-4,5-dihydro-imidazol-1-yl)-pentanoic acid (MG-H2). MG-H2 elutes as 2 peaks, referred to as MG-H2a and MH-H2b. AGE levels were standardised against Ser to compensate for any losses during processing (*e.g.* loss of protein during TCA precipitation and acetone washing steps). In our hands, the MSA hydrolysis method gives good recovery of parent amino acids (>85%, except for Met >75%), and the inter-batch coefficient of variation is typically <8%. AGE recovery experiments with ∼100 µM AGE standards hydrolysed with MSA in the absence of protein gave recoveries of 69±8% for CML, 66±13% for CEL, 45±17% for MG-H1, 52±8% for MG-H2a, and 48±11% for MG-H2b (*cf*. 40–75% for HCl hydrolysis [24]). Whilst these recoveries are lower than those reported using exhaustive enzymatic digestion (*cf*. 75–96% recoveries for the same AGEs [25]), acid hydrolysis avoids the potential for altered digestion efficiencies/missed cleavages at modified residues, and does not require the addition of a large amount of protein in the form of multiple proteases, which must be corrected for in order to arrive at the reported recoveries. The limits of detection for the AGEs were ∼0.4 pmol for CML (∼9 µmol/mol Ser), ∼0.25 pmol for CEL (∼6 µmol/mol Ser), ∼0.6 pmol for MG-H1 and MG-H2a (∼13 and 14 µmol/mol Ser respectively), and ∼0.8 pmol for MG-H2b (∼18 µmol/mol Ser).

### Statistical Analysis

Statistical analyses were performed using GraphPad Prism (ver. 5 for Macintosh; GraphPad Software, La Jolla, CA, USA), with *P*<0.05 taken as significant. Correlation analysis for MGO-dependent lysate enzyme inhibition was by Pearson correlation, with one-tailed *P*-values reported. Specific statistical tests are reported in the Figure legends.

## Results

### Effect of Reactive Aldehydes and Glucose on Total Thiol Concentrations in HCAEC

**Figure 2:**
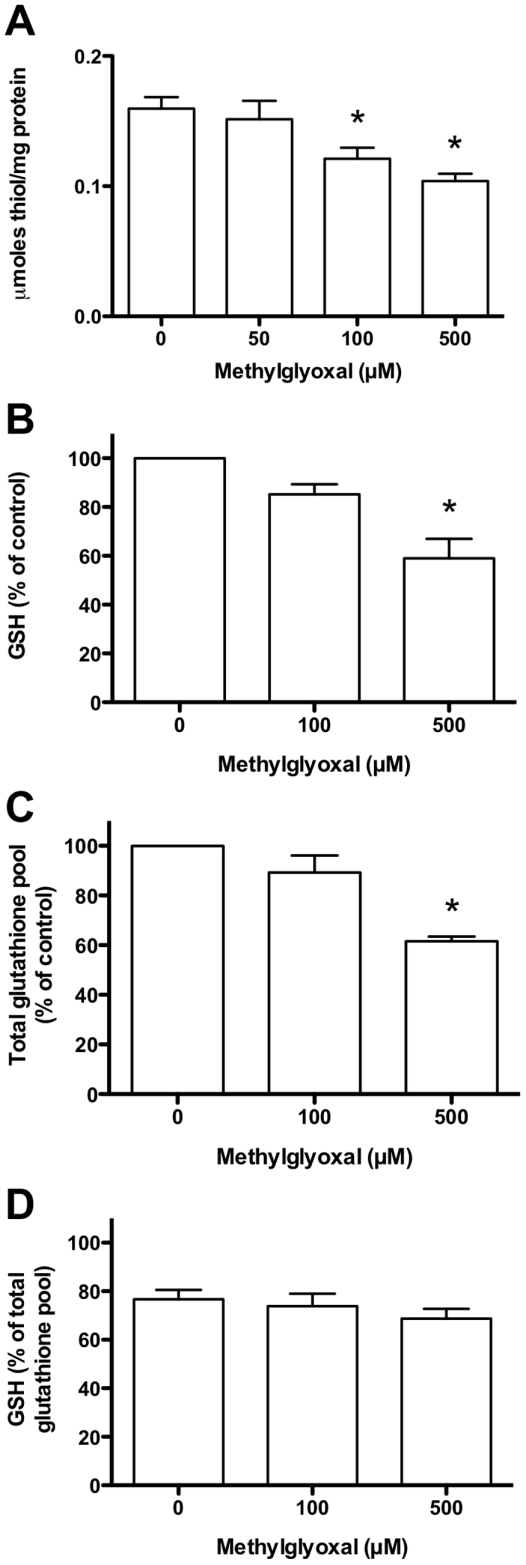
Modulation HCAEC thiol and glutathione levels by MGO. HCAEC were incubated with 0–500 µM MGO in serum-free medium for 1 h at 37°C, prior to lysis and analysis. (A) Total thiols (protein plus low molecular mass) normalised to protein content. (B) GSH levels as a % of the 0 µM MGO control concentration. (C) Total glutathione pool (GSH+GSSG+protein-SSG) as a % of the 0 µM MGO condition. (D) GSH expressed as a % of the total glutathione pool (GSH+GSSG+protein-SSG). Data are means+SEM of ≥3 independent experiments, each performed in triplicate. *Significant change (*P*<0.05) compared to 0 µM control (one-way ANOVA with Tukey’s post-test, using repeated measures in (A), and log-transformed data in (B–D)).

Total thiol concentrations (protein plus low-molecular-mass) standardised to cell protein were used to assess changes in redox status of the HCAEC exposed to glucose or reactive aldehydes. HCAEC (2–3×10^5^ cells/well) were incubated with MGO, glyoxal or glycolaldehyde (0–500 µM, 1 h, 37°C) in HBSS, or glucose added to cell media (25 mM, 24 h, 37°C). These treatments did not alter cell viability or protein levels compared to controls (data not shown). Treatment with 100 or 500 µM MGO resulted in significant decreases (∼35%) in total thiols compared to controls (Figure 2A). Treatment with up to 500 µM glyoxal or glycolaldehyde, or elevated glucose (25 mM) did not cause significant changes (data not shown). As a consequence subsequent experiments examined only MGO.

### Effect of MGO on Reduced and Oxidised Glutathione

HCAEC (1×10^4^ cells/well) were incubated as above, and analysed for GSH, GSSG/protein-SSG, and the ratio of these species. 500 µM MGO induced a significant loss of GSH (∼40%) compared to controls (Figure 2B). A significant decrease was also detected for the total glutathione pool (GSH+GSSG+protein-SSG; Figure 2C). When GSH levels were expressed as a percentage of the total pool (Figure 2D) there was a trend towards a more oxidising environment (less GSH, more GSSG) with increasing MGO (77±8% present as GSH in control cells; 69±7% with 500 µM MGO), however this did not reach statistical significance.

### Effect of MGO Treatment on NADP(H)

**Figure 3:**
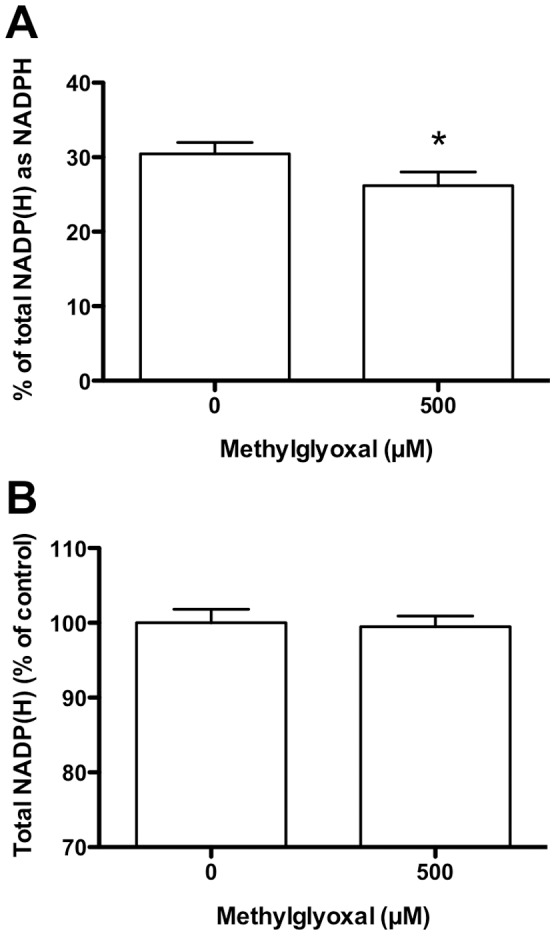
NADPH levels in HCAEC following incubation with MGO. HCAEC were incubated with 0–500 µM MGO in serum-free medium for 1 h at 37°C, prior to lysis and analysis. (A) Percentage of NADP(H) that is present in the reduced form (NADPH). (B) Total NADP(H) pool (NADP^+^+NADPH). Data are means+SEM of 3 independent experiments each performed in triplicate. *Significant change (*P*<0.05) compared to 0 µM control (paired *t*-test of log-transformed data).

Exposure to 500 µM MGO resulted in a significant decrease (∼10%) in the concentration of NADPH (Figure 3A), but no significant change in the total pool (NADP^+^+NADPH) (Figure 3B).

### Effect of MGO Treatment on NADPH Generating Enzyme Activities in Intact HCAEC

**Figure 4:**
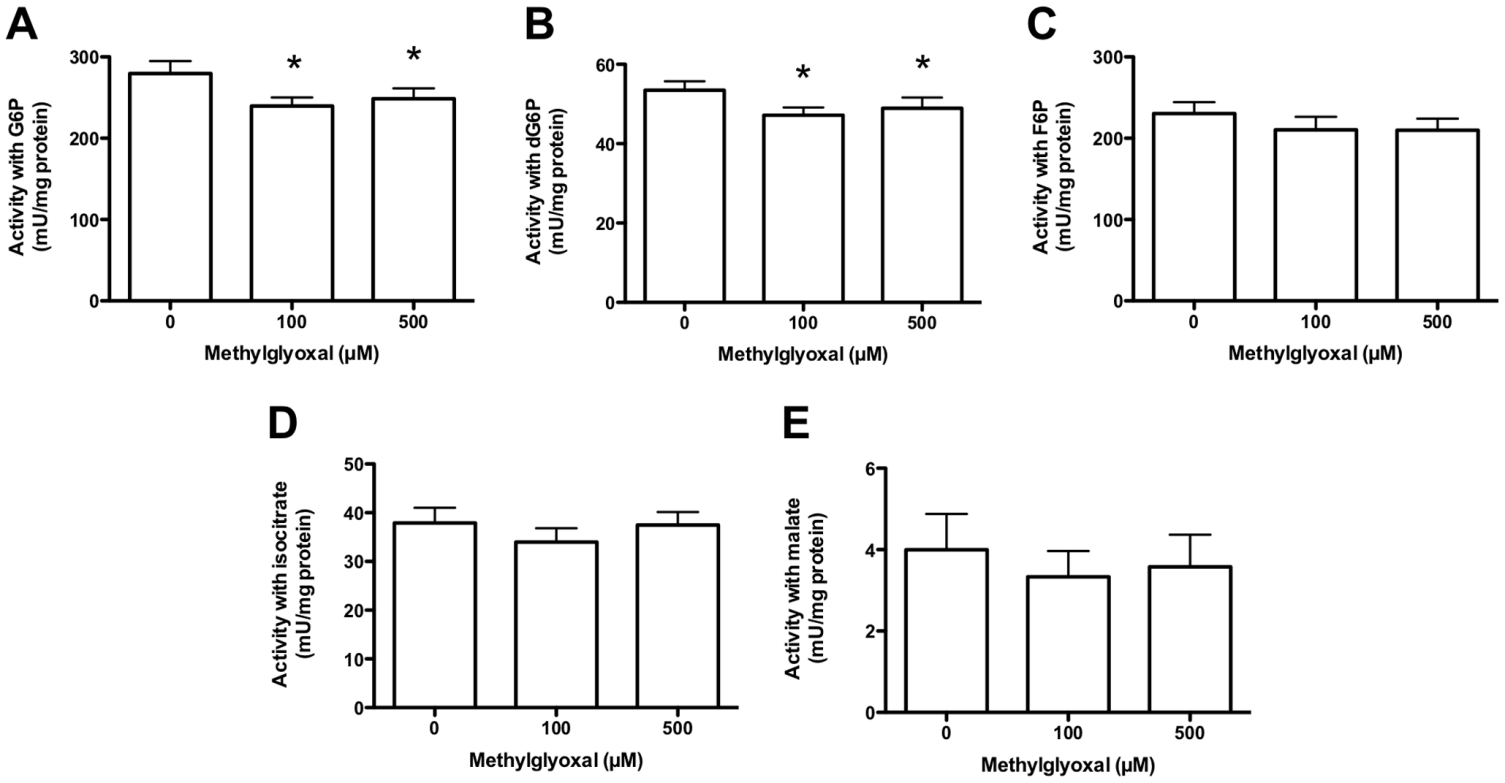
Activity of NADPH-producing enzymes in HCAEC following incubation with MGO. Intact HCAEC (2–3×10^5^ cells/well) were incubated with 0–500 µM MGO for 1 h in serum-free medium, with enzymatic activities (measured as conversion of NADP^+^ to NADPH) determined with the following substrates after lysis: (A) glucose 6-phosphate, G6P; (B) 2-deoxyglucose-6-phosphate, dG6P; (C) fructose 6-phosphate, F6P; (D) isocitrate, or; (E) malate. Data are means+SEM of ≥3 experiments, each performed in triplicate. *Significant change in activity (*P*<0.05) compared to 0 µM control activity (one-way ANOVA with Tukey’s post-test).

HCAEC were incubated with MGO (as above), followed by lysis and measurement of NADPH production from NADP^+^ in the presence of substrates for various enzymes. With G6P as substrate, MGO-treatment resulted in a significant loss (∼11% at 500 µM MGO; Figure 4A) of NADPH-producing enzyme activity in cells exposed to 100 or 500 µM MGO compared to 0 µM MGO). This activity is a composite of that from multiple enzymes of the pentose phosphate pathway including G6PD (EC 1.1.1.49, the first enzyme in the pathway), 6-phosphogluconate dehydrogenase (6PGD, EC 1.1.1.44, the third enzyme in the pathway; assuming that gluconolactonase, EC 3.1.1.17, the second enzyme in the pathway, is intact), and H6PD (EC 1.1.1.47) as this can also use G6P [26].

H6PD activity was assayed under identical conditions using dG6P; this sugar is not metabolised by G6PD [26]. Significant inhibition was observed with 100 and 500 µM MGO compared to controls (∼9% at 500 µM MGO; Figure 4B). With fructose-6-phosphate (F6P) as substrate, the combined activity of G6PD, 6PGD and H6PD was detected, as a result of the rapid conversion of F6P to G6P (a reversible, non-rate-limiting, reaction at physiological substrate concentrations) by phosphoglucose isomerase (EC 5.3.1.9) present in human endothelial cells (Figure 1). In this case, up to 500 µM MGO did not decrease NADPH production (Figure 4C).

The combined NADPH-generating activity of mitochondrial and cytosolic IDH (EC 1.1.1.42) was assessed using isocitrate. No loss of IDH activity was detected under identical conditions to those described above (Figure 4D). With malate as substrate (*i.e.* total activity of mitochondrial and cytosolic ME; EC 1.1.1.40) MGO treatment (as above) did not reduce NADPH formation (Figure 4E).

### Effect of MGO on HCAEC Lysate Activities

The above data are consistent with decreased activity of multiple NADPH-producing enzymes, however only short incubation periods could be examined using intact cells due to the absence of cell growth medium; this was omitted to prevent MGO from reacting with the amino acids and proteins present in media. To further examine the reactions of MGO with HCAEC, additional experiments were conducted with lysates, in which longer incubation times (3 h at 37°C) and higher concentrations (up to 5 mM) of MGO could be utilised.

**Figure 5:**
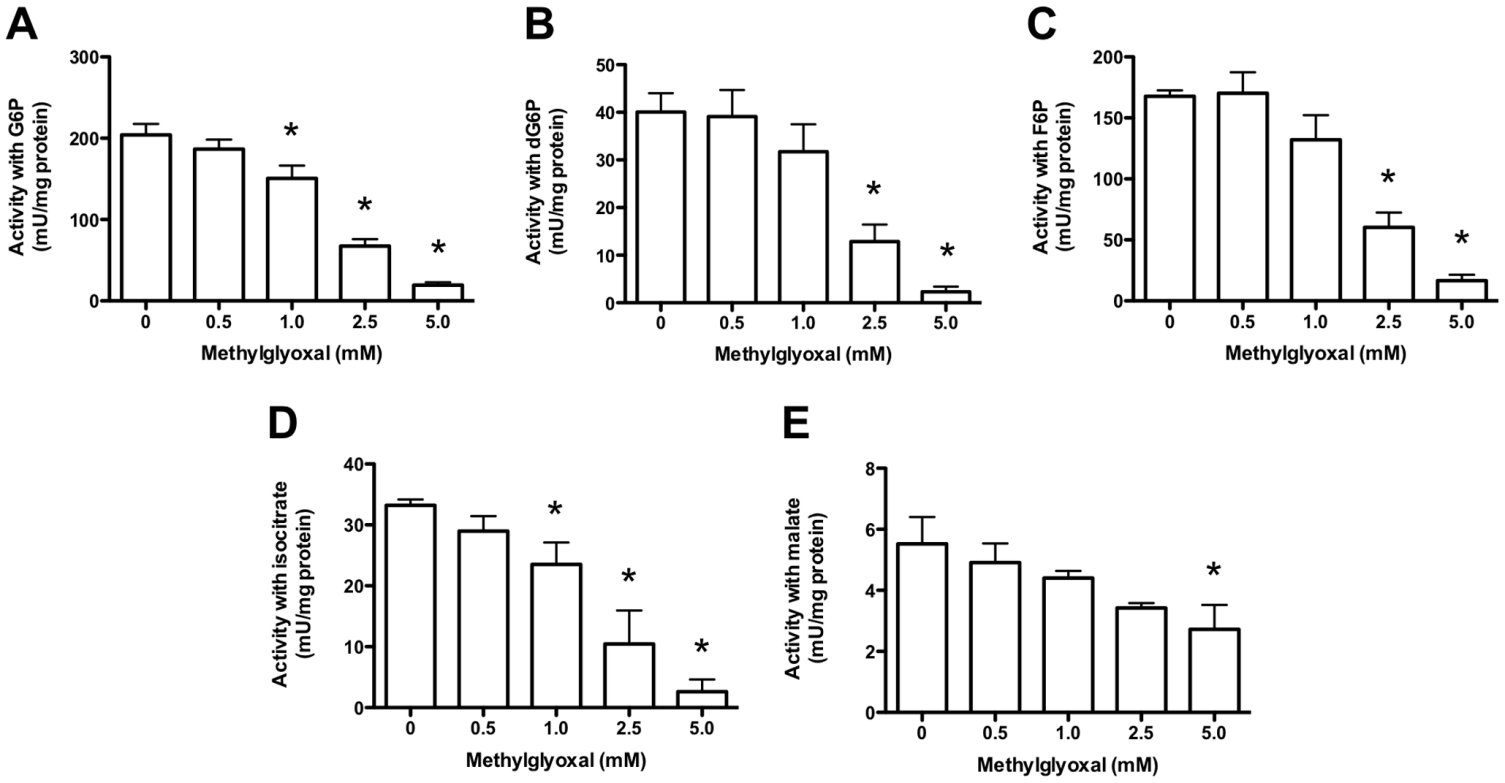
Activity of NADPH-producing enzymes in HCAEC lysates incubated with MGO. HCAEC lysates (1 mg protein/mL) were incubated for 3 h with 0–5 mM MGO. Activity was determined with the substrates: (A) G6P; (B) dG6P; (C) F6P; (D) isocitrate, or; (E) malate. Data are means+SEM of ≥3 experiments, each performed in triplicate. *Significant change in activity (*P*<0.05) compared to 0 mM control activity (one-way ANOVA with Tukey’s post-test).

Incubation of lysates (1 mg protein/mL) with MGO (0–5 mM, 3 h at 37°C), followed by assay with the same substrates used for the intact cells, resulted in dose-dependent inhibition, with good linearity up to 2.5 mM MGO, followed by a slight decrease in the rate of inhibition above 2.5 mM. Use of G6P as substrate (combined G6PD, 6PGD and H6PD activity) resulted in dose-dependent inhibition of NADPH generation (Pearson *R* ^2^ = 0.9945 over the range 0–2.5 mM MGO; one-tailed *P* = 0.0014), which reached significance at ≥1 mM MGO (Figure 5A). Use of dG6P to measure H6PD activity (Figure 5B; *R* ^2^ = 0.9730; *P* = 0.0068), or F6P to measure G6PD +6PGD+H6PD activity (Figure 5C; *R* ^2^ = 0.9567; *P* = 0.0110), both showed inhibition that was statistically significant with ≥2.5 mM MGO. With isocitrate as substrate (mitochondrial and cytosolic IDH activity) significant enzyme dose-dependent inhibition (*R* ^2^ = 0.9985; *P* = 0.0004) was observed with ≥1 mM MGO (Figure 5D), whilst activity with malate as substrate (mitochondrial and cytosolic ME activity) was significantly decreased only at 5 mM MGO (Figure 5E; *R* ^2^ = 0.9763; *P* = 0.0060).

The effect of lysate protein concentration on MGO-derived inhibition was examined with G6P, isocitrate and malate as substrates. With higher protein levels (2 mg/mL), significant inhibition of NAPDH formation, using G6P or isocitrate as substrate, was observed with ≥2.5 mM and 5 mM MGO respectively. No inhibition was observed with malate as substrate at these MGO levels (data not shown). These data indicate that the extent of loss of NAPDH generating activity is dependent on both protein and MGO concentrations.

### Detection of AGE Adducts in Intact HCAEC and Lysates Treated with MGO

**Figure 6:**
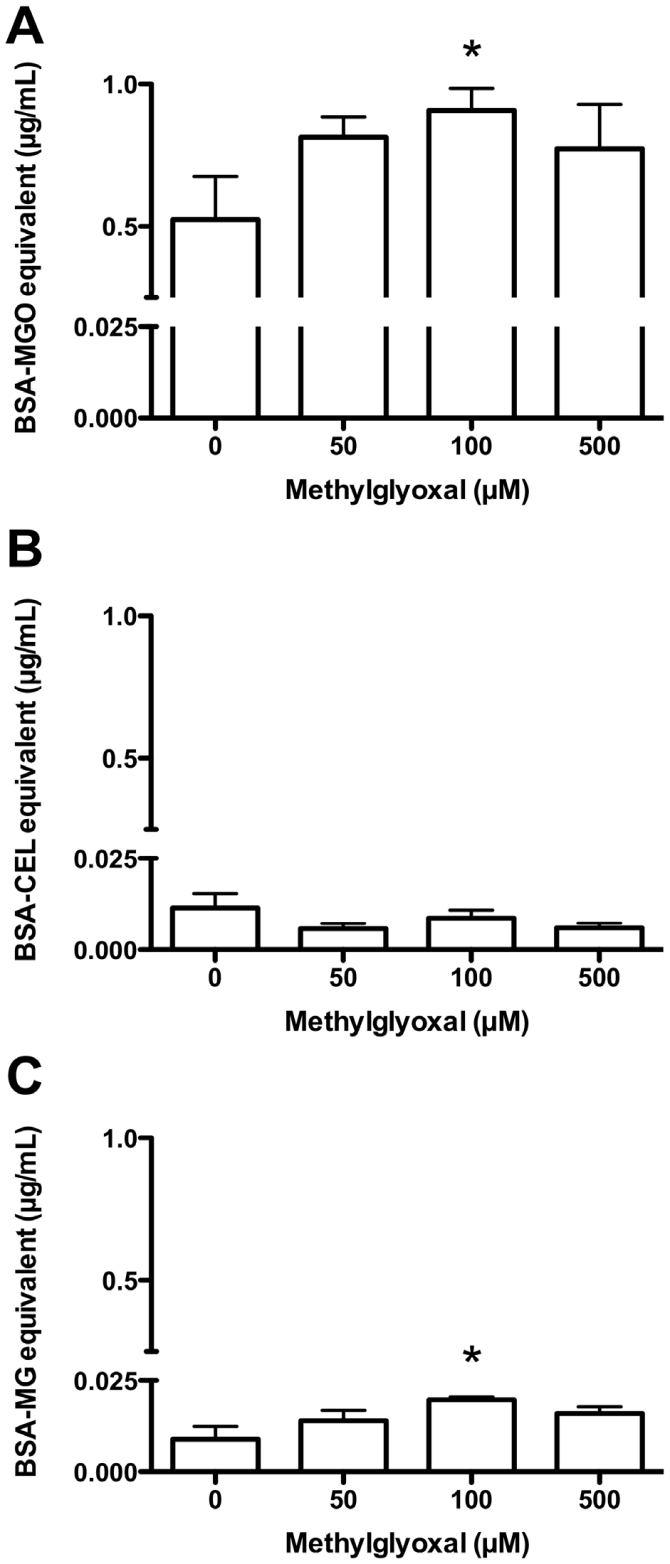
AGE formation on intact HCAEC following incubation with MGO. Intact HCAEC (3×10^5^ cells/well; 2–3 wells per donor) were incubated with 0–500 µM MGO for 1 h in HBSS, with the formation of AGE determined by ELISA after cell lysis: (A) hydroimidazolones; (B) CEL, and; (C) CML. Data are means+SEM of AGE formation for 4 HCAEC donors, measured in duplicate ELISA wells. *Significant change in activity (*P*<0.05) compared to 0 mM control activity (one-way ANOVA with Tukey’s post-test).

Incubation of intact HCAEC with MGO as per the enzyme activity experiments (0–500 µM, 1 h at 37°C) showed significant hydroimidazolone formation compared to controls, which was dependent on the MGO concentration and maximal at 100 µM MGO (0.907±0.156 µg/mL BSA-MGO equivalents; Figure 6A). Control levels of CEL were low (0.011±0.008 µg/mL BSA-CEL equivalents), and levels did not increase at any of the MGO concentrations examined (Figure 6B). CML levels followed a similar trend to the hydroimidazolones (Figure 6C), but were present at ∼80-fold lower concentrations, based on the assumption of equal glycation of the BSA-MGO and BSA-CML standards (0.020±0.001 µg/mL BSA-CML equivalents at 100 µM MGO).

**Figure 7:**
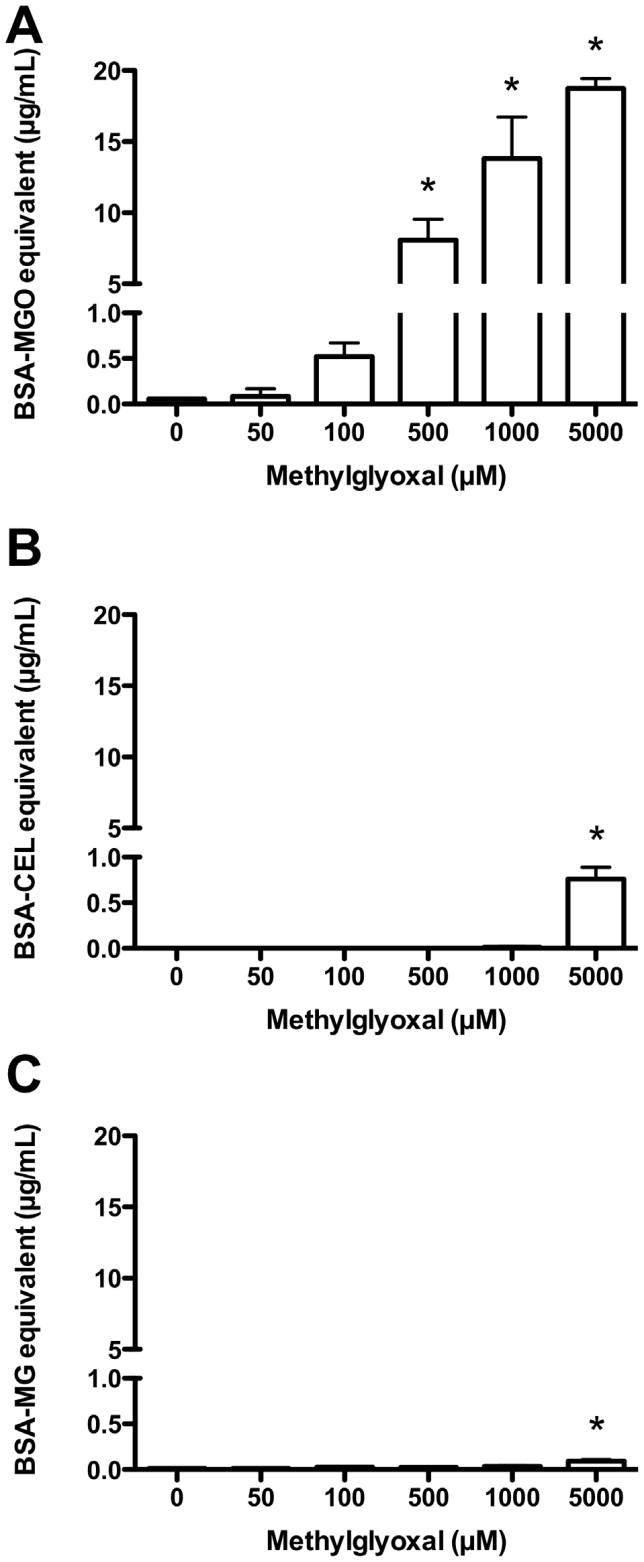
AGE formation on HCAEC lysates following incubation with MGO. Pooled HCAEC lysates from 4 donors (1 mg protein/mL) were incubated with 0–5 mM MGO for 1 h in PBS, with the formation of AGE determined by ELISA after cell lysis: (A) hydroimidazolones; (B) CEL, and; (C) CML. Data are means+SEM of AGE formation for 3 independent incubations, each measured in duplicate ELISA wells. *Significant change in activity (*P*<0.05) compared to 0 mM control activity (one-way ANOVA with Tukey’s post-test).

Hydroimidazolones were also the major MGO-derived protein glycation products detected in experiments where HCAEC lysates were incubated, as in the enzyme activity studies, with MGO (0–5 mM, 3 h at 37°C; Figure 7A). Hydroimidazolone levels did not reach a plateau as seen with the intact HCAEC, instead increasing with as MGO concentrations increased. Hydroimidazolone levels were significantly elevated (*P*<0.05) compared to the 0 µM control with ≥500 µM MGO. Similar statistical analyses in the absence of the ≥500 µM data showed a significant increase at 100 µM MGO. In contrast, significant increases in CEL (Figure 7B) and CML (Figure 7C) were only evident at 5 mM MGO (0.760±0.076 µg/mL BSA-CEL equivalents; 0.093±0.020 µg/mL BSA-CML equivalents); in both cases the overall concentrations were much lower than for the hydroimidazolones (∼25- and ∼200-fold lower, respectively).

### Detection of AGE Adducts in MGO-treated Lysates by HPLC

**Figure 8:**
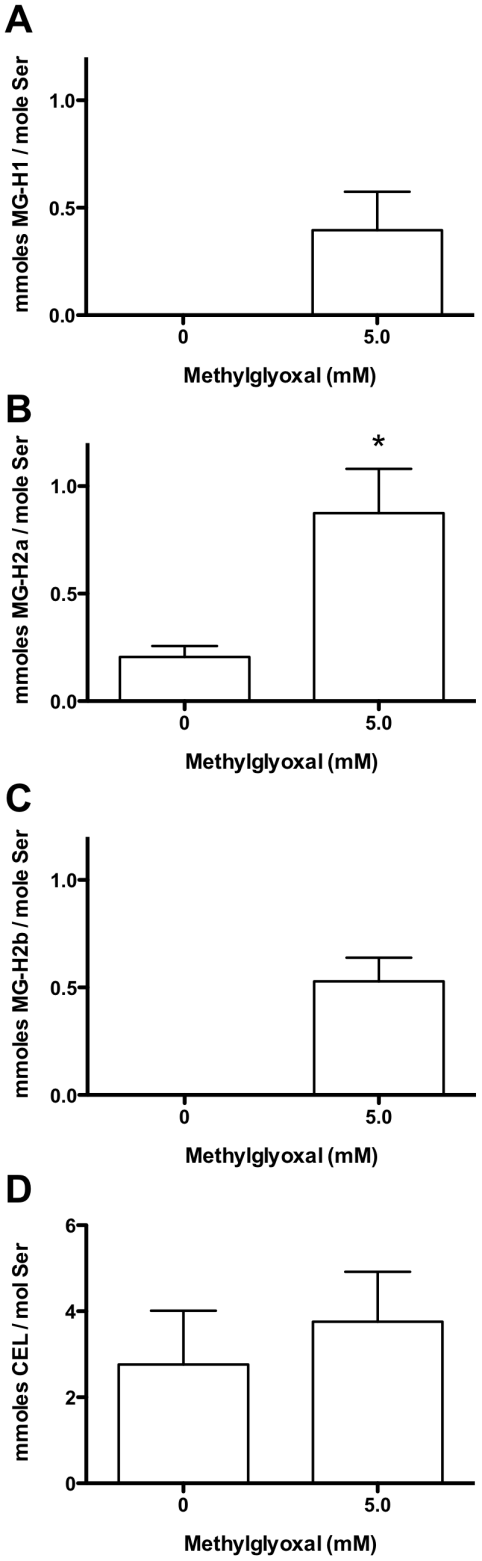
HPLC identification and quantification of AGE formation on HCAEC lysate proteins following incubation with MGO. HCAEC lysates (1 mg protein/mL) were incubated for 3 h with 0–5 mM MGO, followed by HPLC separation and analysis. (A) MG-H1, (B) MG-H2a, (C) MG-H2b, (D) CEL. Data are means+SEM of 3 independent experiments performed in duplicate. Statistical analysis was by paired *t*-test for (B) and (D), and Wilcoxon’s sign-ranked tests for (A) and (C), as the control values were below the limit of detection (∼13 µmol/mol Ser for MG-H1, and ∼18 µmol/mol Ser for MG-H2b), and must therefore be considered non-Gaussian. *Significant change (*P*<0.05) compared to 0 mM control.

In order to confirm the identities of the major MGO-derived glycation products, HCAEC lysates (1 mg protein/mL) were incubated with MGO (0 or 5 mM, 3 h at 37°C), followed by acid hydrolysis and HPLC analysis of AGE formation on total proteins. As this method is not as sensitive as the ELISA-based methods, only the highest MGO concentration was examined. Arg-derived hydroimidazolones were the major protein glycation product detected in the samples treated with 5 mM MGO, but not the controls (Figure 8). A significant increase in MG-H2a (Figure 8B), but not MG-H1 (Figure 8A) or MG-H2b (Figure 8C), was observed with MGO treatment; control (0 mM MGO) levels of MG-H1 and MG-H2b were below the detection limit. Absolute CEL levels were higher than hydroimidazolone levels, however this was mainly due to background levels and/or a co-eluting peak in the lysate chromatograms; furthermore, there was no significant difference between control and MGO-treated samples (Figure 8D). CML and MOLD levels could not be determined due to interference.

## Discussion

The results reported here show that the cellular redox balance of intact human coronary artery endothelial cells (HCAEC) is altered by incubation with bolus micromolar doses of MGO for relatively short time periods. This was demonstrated by a decrease in total reduced thiols (protein plus low-molecular-mass), GSH and NADPH, with these changes not resulting in any immediate loss of cell viability. In contrast to MGO, incubation with glyoxal, glycolaldehyde, or levels of glucose seen in the blood of people with poorly controlled diabetes, did not induce significant changes in cellular redox status under the conditions used. MGO inhibited the activity of multiple intracellular enzymes that convert NADP^+^ to NADPH, under identical conditions, providing a rationale for the decreased NADPH and reduced thiol levels. Further experiments with both MGO-treated intact cells and cell lysates demonstrated that MGO-mediated hydroimidazolone formation on Arg residues may account for the observed inhibition of NADPH-generating enzymes.

The bolus micromolar MGO concentrations and short incubation times used in the experiments reported here represent a likely difference to *in vivo* conditions, where the endothelium would be expected to be exposed to a continual external flux of MGO over an extended time period. Although it would be possible to perform studies using longer term exposures to lower levels of MGO *via* continual infusion, this would necessitate maintaining the cells in media, and result in considerable additional complexities, as the proteins and other materials in media are a competing target for the MGO, rather than the cells alone as in the studies reported here.

Further, although the MGO concentrations used were higher than reported plasma steady-state levels (which are generally considered to be low micromolar at most [5], though some studies suggest up to ∼400 µM MGO [27]), MGO concentrations in cells and tissues, such as within the artery wall, may be significantly greater than these plasma values, as a result of intracellular MGO production *via* increased triosephosphate formation (glycolytic metabolism, the Embden-Meyerhof pathway) and subsequent degradation [28]. To this end, MGO levels have been reported to be 20-fold high in the eye lens than in plasma [29]. The concentrations employed in the intact cells studies reported here might therefore be within the concentration range to which cellular proteins are exposed *in vivo*.

It should be noted that the reported plasma levels reflect steady-state concentrations (*i.e.* residual material that has not reacted with plasma components), rather than the *total* concentration to which proteins are likely to be exposed to over their biological lifetime. MGO has a plasma lifetime of minutes at most, on the basis of the experimental rate constant for the initial reaction of methylglyoxal with *N*-acetylarginine residues (reported as 8.5×10^−3^ M^−1^ s^−1^ in [30]). These data yield a half-life for MGO of ∼80 s. Thus cellular proteins with half-lives of hours to days are likely to be exposed to a total flux of MGO which is orders of magnitude greater than the *in vivo* plasma steady-state levels.

With regard to the modifications to the cellular redox balance observed in the current HCAEC studies, the decrease in total thiols observed on MGO treatment is consistent with direct adduction of MGO to thiols (*cf.* [23]) and/or the induction of oxidative stress, as thiol loss is a non-specific marker of oxidation/modification [22]. GSH loss would be expected to increase oxidative stress, as the activities of GPx and Grx require GSH and ultimately NADPH (*cf*. Figure 1), thus potentially decreasing the ability of HCAEC to survive further oxidative stress.

The parallel loss of GSH and total glutathione (GSH+GSSG) is consistent with removal of both oxidised and reduced glutathione, rather than conversion of GSH to GSSG. This may be due to GSH consumption by MGO that exceeds the capacity of the glyoxalase system to detoxify MGO and regenerate GSH. The observed decrease in NADPH may exacerbate this effect, as GSH regeneration from GSSG is NADPH dependent. In addition to perturbing GSH levels, decreased NADPH may adversely affect the Trx/TrxR and Prx systems, which require NADPH (Figure 1). Consistent with this, it has been reported that Trx concentrations and TrxR activity are decreased in both human [31] and bovine [32] aortic endothelial cells treated with MGO. Perturbation of the Trx/TrxR system would be expected to decrease Prx-mediated oxidant (H_2_O_2_, hydroperoxides, peroxynitrite) removal, as the Prx family utilise Trx for reducing equivalents; indeed this has been shown in human aortic endothelial cells [31], albeit with considerably higher MGO concentrations than observed under physiological conditions (1–5 mM).

NADPH is also an essential cofactor for multiple processes relevant to cardiovascular disease, endothelial dysfunction and diabetes, including fatty acid and cholesterol synthesis, nitric oxide production (*via* nitric oxide synthases) and superoxide radical formation (by NADPH oxidases) [10]. Decreased NADPH levels may result from both deceased generation from NADP^+^ and/or increased consumption by anti- and pro-oxidant enzymes such as eNOS and NOX [33]. However, the observed inhibition of multiple NADPH-generating enzyme activities by MGO supports decreased NADPH-generating capacity being an important contributing factor. Further, unlike the GSH pool, the total (NADP^+^+NADPH) pool did not decrease (Figure 3B). With G6P (a substrate for G6PD, 6PGD and H6PD), and dG6P (a substrate for H6PD) significant NADPH-generating enzyme inhibition was observed, consistent with enzyme modification. In contrast, with F6P as substrate (total of G6PD +6PGD+H6PD activities) the activity loss was not significant; this difference may be due to the conversion of F6P to G6P by phosphoglucose isomerase being a limiting process following MGO treatment, rather than metabolism of G6P by G6PD/H6PD (Figure 1). High fructose levels have been shown to inhibit G6PD activity, with this being implicated in lens cataractogenesis [34]. In the current study, intact HCAEC experiments showed no significant loss of IDH or malic enzyme activity using isocitrate and malate as substrates, respectively, whereas with the cell lysates inhibition of both IDH and malic enzyme was detected at high MGO levels.

In support of the current data, studies of rat liver and pancreas indicate that hyperglycaemia results in reduced G6PD activity, with this accompanied by a reduced NADPH/NADP^+^ ratio, and decreased GSH levels [35]. Studies of adult cardiomyocytes have also shown an association between G6PD inhibition, GSH loss, and contractile dysfunction, with this proposed to occur *via* NADPH depletion [36]. Similarly, knock-out of G6PD in vascular smooth muscle has been reported to result in decreased NADPH levels, consistent with this enzyme being a key regulator of cellular NADPH levels [37]; this conclusion is supported by studies on genetic deficiencies (*e.g.* [38]). Although the above studies suggest that G6PD is the key enzyme responsible for maintaining cellular NADPH levels, this is not universally agreed upon; for example, studies with NIH 3T3 mouse fibroblast cells suggest that IDH, rather than G6PD, is the critical enzyme in that cell type [39].

The exact mechanism of loss of NADPH-generating activity remains to be determined. Activation of cells *via* the AGE/RAGE pathway is known to result in multiple changes that could conceivably result in enzyme inhibition, including induction of oxidative stress and inflammatory cytokines [17]. However, this pathway is unlikely to be the cause of the cellular changes observed in the current experiments, since the intact HCAEC incubations with MGO were carried out in HBSS, thus precluding the generation of extracellular AGEs that could bind to RAGE. In contrast, it is well documented that the reaction of MGO with proteins results in the generation of hydroimidazolones on protein Arg residues (reviewed in [9]). In support of this, the ELISA studies examining protein glycation in both intact HCAEC and HCAEC lysates show that MGO-derived enzyme inactivation is paralleled by protein-bound hydroimidazolone formation (confirmed by acid hydrolysis and HPLC to be MG-H1 and MG-H2), even at micromolar MGO concentrations. MGO-derived formation of CEL and CML was 1–2 orders of magnitude lower, consistent with previous studies [40]. Although the analysis of only bulk proteins, rather than specific species, is a clear limitation of the current study (the detection limit of the techniques used prevented examination of specific species), the data obtained suggest that Arg modification on NADPH-producing enzymes may play a role in their inhibition. This hypothesis is supported by previous peptide mass mapping studies of the isolated NADPH-producing enzymes G6PD and IDH that were inhibited by MGO, which identified hydroimidazolone formation on Arg residues that are responsible for NADPH and substrate binding [19]. Whilst evidence has been obtained for significant hydroimidazolone formation (*i.e.* AGEs) from Arg residues, the formation of other materials (both on Arg, and other residues such as Lys and Cys) cannot be excluded, and is likely. The absolute levels of these hydroimidazolones, which are only a few percent for the systems where the lysates were incubated with up to 5 mM MGO (even if hydroimidazolone loss during processing is corrected for and assuming a Ser: Arg ratio of 1∶1), are likely to markedly underestimate the true level of adduction, as these are formed *via* multiple steps (some of which are reversible) and involve a number of undetected intermediates.

In summary, the data obtained in this study partially support our initial hypothesis that elevated glucose and reactive aldehydes can modulate the redox balance of endothelial cells *via* inhibition of NADPH-generating enzymes, resulting in decreased reduced thiol and NADPH levels. MGO, but not glyoxal, glycolaldehyde, or high glucose, was able to readily alter the redox state of human coronary artery endothelial cells, as shown by significantly decreased reduced cell thiol levels. Further experiments with MGO showed (in addition to the thiol data) decreased cellular GSH and NADPH, decreased activity of several NADPH-generating enzymes, and increased protein glycation, thus supporting the role of MGO in modulating cellular redox status *via* inhibition of NADPH-generating enzymes. Hydroimidazolone formation on Arg residues provides at least a partial rationale for the decreased NADPH-generating capacity. These changes may contribute to the endothelial cell dysfunction observed in diabetes-associated atherosclerosis; this could be confirmed in future work by comparing the changes observed in the current study to endothelial function (*e.g.* by monitoring nitric oxide generation). Experiments to elucidate the mechanism(s) responsible for the decreased NADPH-generating capacity could include silencing the various NADPH-generating enzymes and examining the effects on cellular redox status. The effects of transiently elevated MGO levels on endothelial cell function/dysfunction could also be examined, by measuring the parameters examined in this study in a time course following removal of the MGO and continued cell culture. This would help to determine whether MGO may be contributing to the post-prandial endothelial dysfunction observed in people with diabetes [41].

The effects of MGO observed in this study are likely to be most relevant in situations where large fluctuations in blood glucose levels occur, such as in people with uncontrolled diabetes or ketosis, since chronic hyperglycaemia and reactive aldehyde exposure are known to cause compensatory increases in both the protein level, and activities, of enzymes including glyoxalase I, aldose reductase, peroxiredoxins 1 and 3, glutathione peroxidase 1, and superoxide dismutase-1 [42], [43]. However, this mechanism is likely to play a role even in people with well-controlled diabetes, since the elevated MGO levels observed in these people [4] suggests that the glyoxalase system in not able to fully compensate. It should also be remembered that undiagnosed or poorly controlled diabetes is an increasing problem worldwide, particularly since diabetes has emerged as a major health problem in developing countries [44], where millions of people with diabetes are likely to have limited access to high quality health care.
